# P-2167. Outcomes of Nocardiosis Among Immunosuppressed Patients

**DOI:** 10.1093/ofid/ofaf695.2330

**Published:** 2026-01-11

**Authors:** Maria Vega Brizneda, Cyndee Miranda, Eric Cober, Anisha Misra, Susan Harrington, Zachary Yetmar

**Affiliations:** Cleveland Clinic, Cleveland, OH; Cleveland Clinic, Cleveland, OH; Cleveland Clinic Foundation, Cleveland, OH; Cleveland Clinic Foundation, Cleveland, OH; Cleveland Clinic, Cleveland, OH; Cleveland Clinic, Cleveland, OH

## Abstract

**Background:**

*Nocardia* has a predilection for infection of immunocompromised patients. However, there is little data assessing if nocardiosis varies in presentation and outcomes between different immunosuppressed populations.

**Figure:**
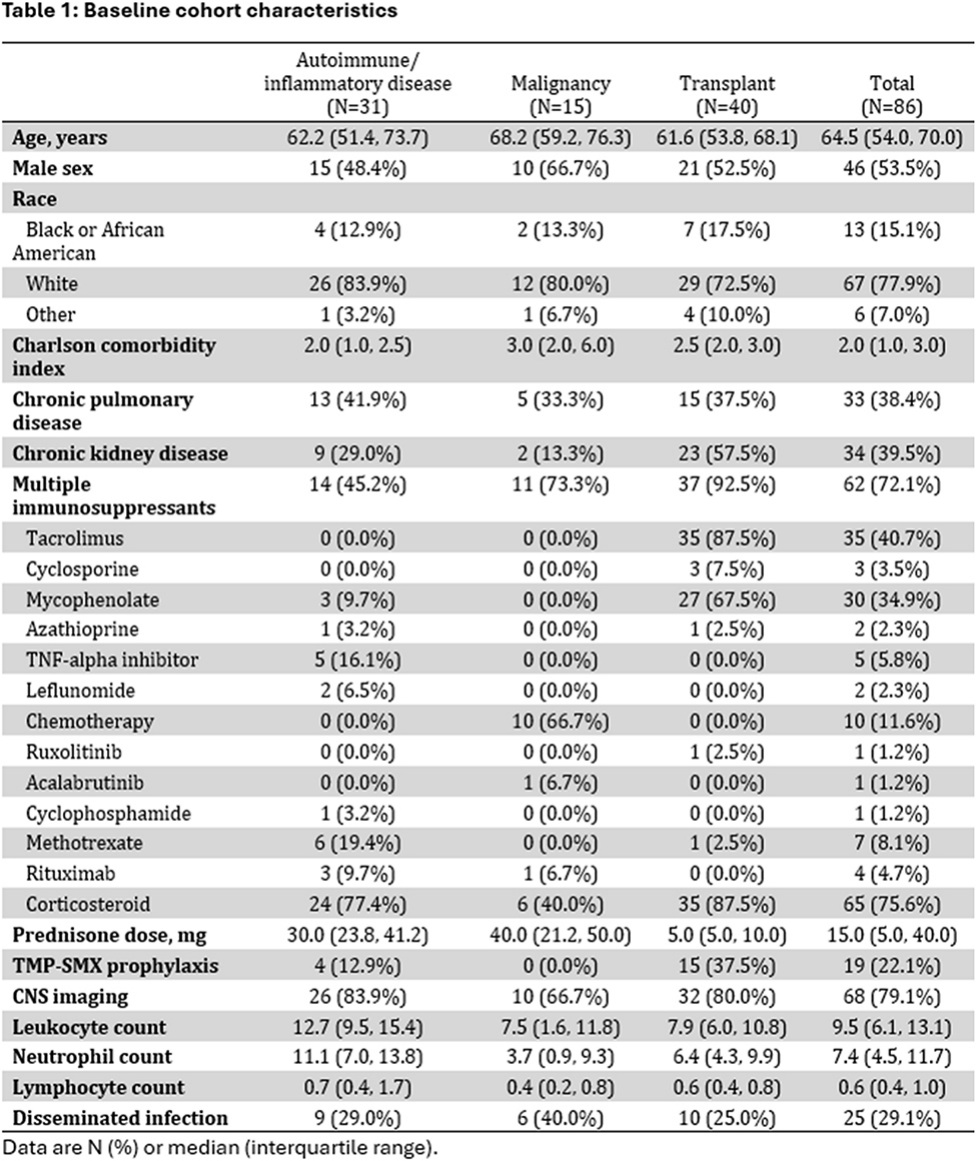

**Figure 1:**
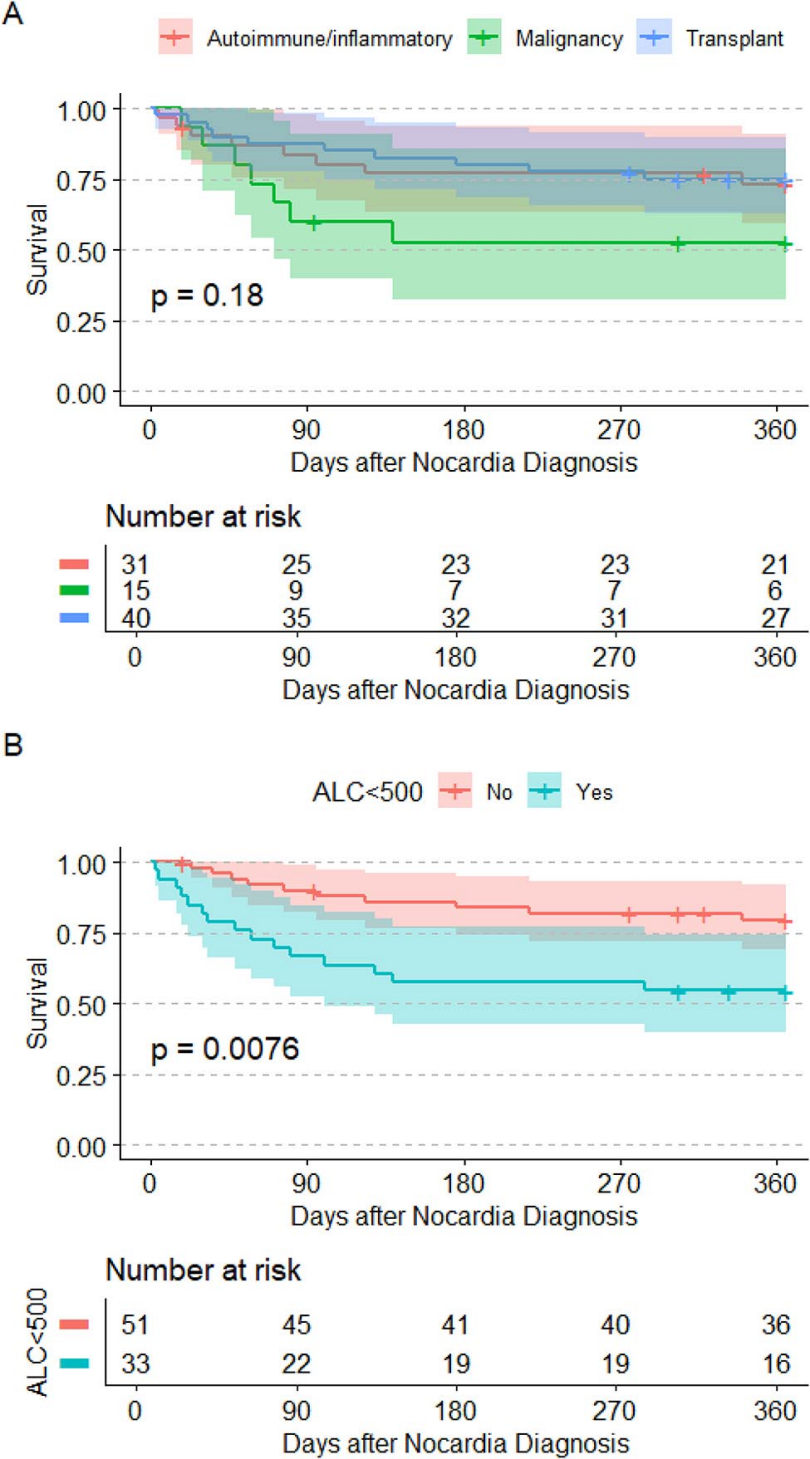
Kaplan-Meier curves of survival after nocardiosis diagnosis between immunosuppression groups (A) and lymphopenia at diagnosis (B).

**Methods:**

We conducted a retrospective cohort study of adult patients diagnosed with nocardiosis from 2010 to 2023 who were prescribed ≥ 20 mg/day of prednisone or any other immunosuppressing medication. Nocardiosis was defined as culture or molecular testing with a *Nocardia* species and compatible signs, symptoms, and/or radiologic findings. Patients were divided by indication for immunosuppression to three groups: transplant (solid organ or stem cell), malignancy, and autoimmune/inflammatory. Dissemination and 1-year mortality were assessed by multivariable logistic and Cox regression, respectively.

**Results:**

86 patients with nocardiosis met criteria and were included. 40 (46.5%) were transplant recipients (including 34 solid organ and 6 stem cell), 15 (17.4%) were immunosuppressed due to malignancy-related therapy, and 31 (36.0%) had autoimmune or inflammatory disease requiring immunosuppression (Table 1). One patient was living with HIV, who was also a kidney transplant recipient. 25 (29.1%) had disseminated infection at presentation, which was similar between groups. Most non-disseminated infections were pulmonary nocardiosis (N=51, 83.6%) and 18 (72.0%) patients with disseminated infection had CNS involvement. 25 (29.1%) also died within 1 year of diagnosis. Neither immunosuppression group or use of multiple immunosuppressants was associated with either dissemination or 1-year mortality (Figure 1). However, lymphopenia (ALC< 500 cells/mL) was associated with dissemination (OR 2.83, 95% CI 1.03-8.08, *p*=0.047) and 1-year mortality (HR 2.58, 95% CI 1.12-5.92, *p*=0.026).

**Conclusion:**

Among a highly immunosuppressed population with nocardiosis, the use of multiple immunosuppressants and indication for immunosuppression did not predict dissemination or mortality. Instead, more functional measures of immunosuppression, such as lymphocyte count, may be more predictive of outcomes in nocardiosis. Future studies should assess other measures of quantitative measures of immune function.

**Disclosures:**

Susan Harrington, PhD, Bruker Daltonics, Inc: Grant/Research Support

